# An Empirical-Mathematical Approach for Calibration and Fitting Cell-Electrode Electrical Models in Bioimpedance Tests

**DOI:** 10.3390/s18072354

**Published:** 2018-07-20

**Authors:** Juan A. Serrano, Gloria Huertas, Andrés Maldonado-Jacobi, Alberto Olmo, Pablo Pérez, María E. Martín, Paula Daza, Alberto Yúfera

**Affiliations:** 1 Instituto de Microelectrónica de Sevilla, Universidad de Sevilla (CSIC-US), Av. Américo Vespucio, 28, 41092 Sevilla, Spain; gloria@imse-cnm.csic.es (G.H); maldonado@imse-cnm.csic.es (A.M.-J.); aolmo@dte.us.es (A.O.); pablopg@imse-cnm.csic.es (P.P.); yufera@imse-cnm.csic.es (A.Y.); 2 Departamento de Electrónica y Electromagnetismo, Facultad de Física, Universidad de Sevilla, Av. Reina Mercedes, SN, 41012 Sevilla, Spain; 3 Departamento de Tecnología Electrónica, Escuela Técnica Superior de Ingeniería Informática, Universidad de Sevilla, Av. Reina Mercedes, SN, 41012 Sevilla, Spain; 4 Departamento de Biología Celular, Facultad de Biología, Universidad de Sevilla, Av. Reina Mercedes, SN, 41012 Sevilla, Spain; mariamartin@us.es (M.E.M.); pdaza@us.es (P.D.)

**Keywords:** bioimpedance, OBT, cell culture, real-time monitoring, microelectrode electrical model, sensing protocol, ECIS

## Abstract

This paper proposes a new yet efficient method allowing a significant improvement in the on-line analysis of biological cell growing and evolution. The procedure is based on an empirical-mathematical approach for calibration and fitting of any cell-electrode electrical model. It is valid and can be extrapolated for any type of cellular line used in electrical cell-substrate impedance spectroscopy (ECIS) tests. Parameters of the bioimpedance model, acquired from ECIS experiments, vary for each cell line, which makes obtaining results difficult and—to some extent-renders them inaccurate. We propose a fitting method based on the cell line initial characterization, and carry out subsequent experiments with the same line to approach the percentage of well filling and the cell density (or cell number in the well). To perform our calibration technique, the so-called oscillation-based test (OBT) approach is employed for each cell density. Calibration results are validated by performing other experiments with different concentrations on the same cell line with the same measurement technique. Accordingly, a bioimpedance electrical model of each cell line is determined, which is valid for any further experiment and leading to a more precise electrical model of the electrode-cell system. Furthermore, the model parameters calculated can be also used by any other measurement techniques. Promising experimental outcomes for three different cell-lines have been achieved, supporting the usefulness of this technique.

## 1. Introduction

In the last few years, many research efforts have been devoted to find a reliable and robust non-invasive technique to estimate and study cell growth on cell-culture assays [1,2,3,4,5] from different viewpoints. Many biomedical setups such as toxicology assays [6], cancer characterization experiments [7,8], biochemical [9], immune-assays [10], stem cells differentiation protocols [11], etc., seek to quantify the number of cells for characterizing a diversity of research objectives and techniques. The Bioimpedance (BioZ)-based measurement approach named ECIS (electrical cell-substrate impedance spectroscopy), senses the electrical response generated on a biological sample, the cell-culture, when it is excited with an alternating current (AC) electrical source, voltage, or current, at several frequencies, as consequence of its conductivity properties. In order to obtain reliable results, the ECIS technique requires precise electronic circuits for picking up the signals of interest [12] as well as accurate electrical models for the electrodes and cell-electrode-solution systems. The aim is to decode the electrical measurements done by the circuits and to express them in terms of the cell number. Some ECIS approaches obtain the impedance response of the cell-electrode system under test without evaluating the electrical model, being the performed impedance signal processing the same for all cell lines or cultures.

Several works on BioZ modelling and monitoring have been reported [4,5,13,14], based on solving the electromagnetic equation involved in the system [5] or Finite Element (FE) simulations [13,14] of the whole cell-electrode-solution system. The obtained results are applied to mono-layer cell-culture configurations, fitting the proposed parameters and/or electrical circuits, to model the cell-electrode-solution. This article proposes a method to tune the electric model for the cell-electrode interface in [13,14], using experimental data gathered from several experiments which were carried out in our research group. Our motivation is mainly derived from the analysis of the parameter evolution observed on our experiments, from the beginning of a cell growth assay, and before, to reach the confluent or mono-layer phase. From this characterization, for subsequent experiments with the same cell-line, it is possible to approximately know the percentage of well filling and the cell density (or number of cells in the well).

In this work, the measurement method is the oscillation-based test (OBT), detailed in [15,16,17,18]. This technique is based on connecting the BioZ as an oscillator. Thus, the oscillation amplitude and frequency vary with the number of cells in the well due to changes in the cell-electrode impedance. This paper details how to know in real time an approximate value of percentage of well filling, using the achieved oscillation amplitude ($a_{osc}$) and frequency ($f_{osc}$) in each moment. To carry out this task, it is necessary to do a previous setting experiment for each cell line. In other words, by means of this initial experiment, a systematic calibration method has been proposed herein for any cell line which uses the OBT approach.

In summary, it is proposed to perform, first, a calibration procedure by employing the OBT technique [15,16,17,18], and second, to validate with this same technique, the results for other assays with different cells densities. Thus, different initial number of cells was seeded to validate the model obtained in the calibration step. Accordingly, a BioZ electrical model of each cell line is obtained, valid for any posterior experiment with this cell line, and leading to a more precise electrical model of the electrode-cell system, useful for any other measurement technique.

## 2. Materials and Methods

### 2.1. Cell-Culture Assay

**Figure A1:**
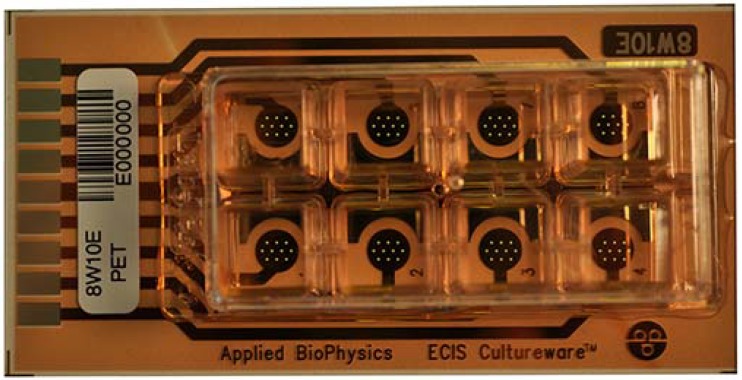
Applied BioPhysics electrodes used.

The employed electrodes for our tests are commercial electrodes of Applied Biophysics [19]. These electrodes contain eight separated wells with ten circular biocompatible gold microelectrodes of 250 μm diameter (Figure A1). The study was carried out in several cell lines, which are described in the following paragraphs.

The first biological sample under testing (SUT) was formed by Chinese hamster ovarian fibroblasts. This cell line is identified as AA8 (American Type Culture Collection). This sample was immersed in McCoy’s medium supplemented with 10% (*v/v*) foetal calf serum; 2 mM l-glutamine, 50 μg/mL streptomycin, and 50 U/mL penicillin. The growing environment was established at 37 °C and 5% CO_2_ in a humid atmosphere. Different initial number of cells were seeded for our experiments: 2500, 5000, and 10,000. Petri plate cultures were also made with the same cell density, to further match with the proposed bio-impedance (BioZ) test.

The second biological samples under testing were two Mouse neuroblastoma (N2a) cell lines. The N2a cell line and its cell line stably expressing wildtype human amyloid precursor protein, N2a-APP, were generously gifted by Prof. Javier Vitorica (Institute of Biomedicine of Sevilla, Sevilla, Spain). Cells were cultured in medium consisting of 50% DMEM High glucose (Biowest, Nuaillé, France) and 50% Opti-MEM (Gibco, Alcobendas, Spain) supplemented with 10% (*v/v*) foetal bovine serum (FBS) (Gibco, Alcobendas, Spain), 2 mM l-glutamine, 50 μg/mL streptomycin and 50 U/mL penicillin (Sigma-Aldrich, Madrid, Spain). N2a-APP was also supplemented with 0.4% Geneticin (Gibco, Alcobendas, Spain). All cell lines were maintained at 37 °C in a humidified atmosphere with 5% CO_2_ and they were routinely subcultured.

In growth curves: the cells were plated in duplicate at 12,500, 6250, and 3125 cells/cm^2^ density in 55 mm dishes and they were allowed to adhere for one day. Dishes were placed at the incubator for 120 h; every 24 h, the cells were trypsinized and counted with a haemocytometer. The cell viability was assessed by a trypan blue exclusion test. Cells from dishes were counted in two independent experiments.

### 2.2. Cell-Electrode Electrical Model

**Figure 1:**
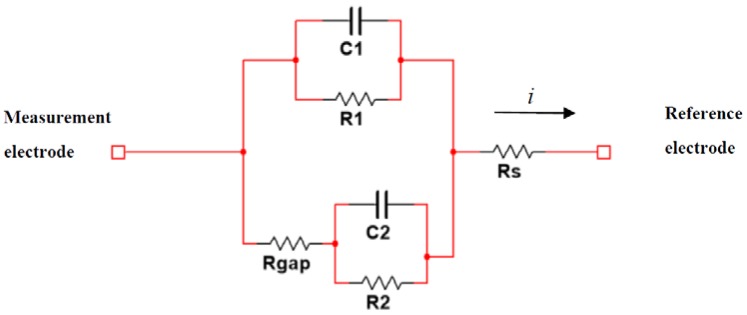
BioZ electric model.

The biological sample under testing was located on a two-electrode system. The first one acted as a reference electrode and the second one was the measurement electrode. Cells were deployed on the electrodes alongside with medium solution. The electrical model describing this cell-electrode interface is presented on Figure 1. The bioimpedance has two poles and two zeros. This model has been widely explored on the literature in [4,5,13]; however, we observed in our experiments that values of electrical parameters changed according to each cell line, and a fitting technique was required to obtain an accurate electrical model in each case. As the OBT technique is used for calibration and fitting, the cell-electrode interface was connected as a load (*Z*) into the proposed oscillator, and, as a result, a biological sensor was created. A start-up signal was provided to the OBT to enable faster measurements and to guarantee the optimal oscillation point for the system, thus avoiding nonlinear behavior from the electrical model. As it was mentioned in the previous section, the variation of the BioZ implied a change on the oscillation values (frequency and amplitude) which were directly related to the number of cells in the culture. This allowed us to measure the cell population and its growth.

The BioZ main electrical-model parameters are *C*, the double-layer capacitance derived from the complex part of the BioZ, and *R*, the transfer resistance representing the biological resistance of the sample. Both elements were placed in parallel [4,13]. The fill-factor parameter (*ff*) was defined as the ratio of the area covered by the cell in the electrodes (if there were no cells, it was 0, and it was 1 when the electrode was fully covered) [15]. For electrodes partially covered by cells, given a *ff* value, the electric model parameters are defined by Equations (1) to (4).

(1) $$R_{1}=\frac{R}{\left(1-ff\right)}$$

(2) $$C_{1}=C\times \left(1-ff\right)$$

(3) $$R_{2}=\frac{R}{ff}$$

(4) $$C_{2}=C\times ff$$

where $C_{1}$ and $R_{1}$ represent the contribution of the empty microelectrodes to the electrical response of the cell culture, and $C_{2}$ and $R_{2}$ depict the electrodes covered by cells. On the other hand, $R_{s}$ models the resistance that the current must overtake to arrive at reference electrode. Finally, $R_{gap}$ represents the equivalent resistance that models the separation zone or interface region between the cell and the electrode.

### 2.3. Model-Oscillation Relationship

Sensitivity curves have been developed to experimentally observe the changes in the BioZ parameters due to the electrical model in constant evolution. In particular, there are two main states: when ff→0 and ff→1 for which the electrical model can be approached by a model with one pole and one zero. From their positions, the other parameters of the model can be obtained using the initial and simplified model in Figure 1. On the other hand, for each cell line, the electrical model will be slightly different, and it will mean that the oscillation parameters of the biological sensor vary. For each cell line, a first experiment was performed to calibrate the electrical model, and in subsequent performed experiments, it was possible to know the number of cells on each well using the oscillation parameters. After carefully studying the model, with the aim of estimating a model for each cell line, we reached the following conclusions:

- ff→0, at the beginning of the experiment:
  ○ The initial frequency can be determined only with the position of the pole, $f_{p}$.
  ○ When the pole location is known, the initial amplitude can be determined using the parameter $R_{s}$. The $R_{s}$ resistance effect is significantly higher in initial amplitude than the effect of $f_{p}$.
  ○ ff→1, at the end of experiment:
  ○ The final frequency and amplitude, at the confluent phase, are highly dependent on the $R_{gap}$ parameter.

**Figure A2:**
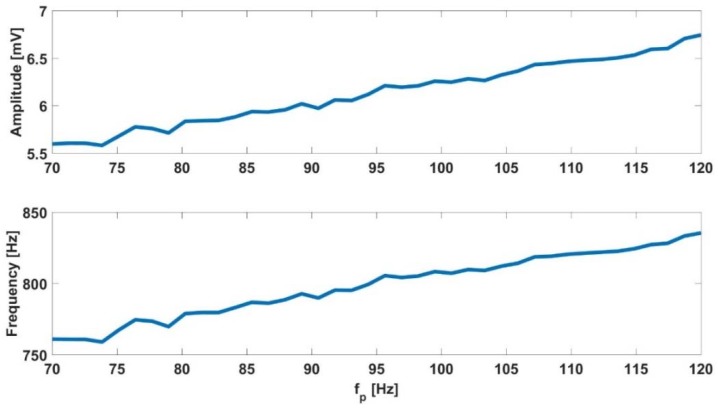
Variation of initial oscillation amplitude and frequency as a function of the pole position *f_p_*.

**Figure A3:**
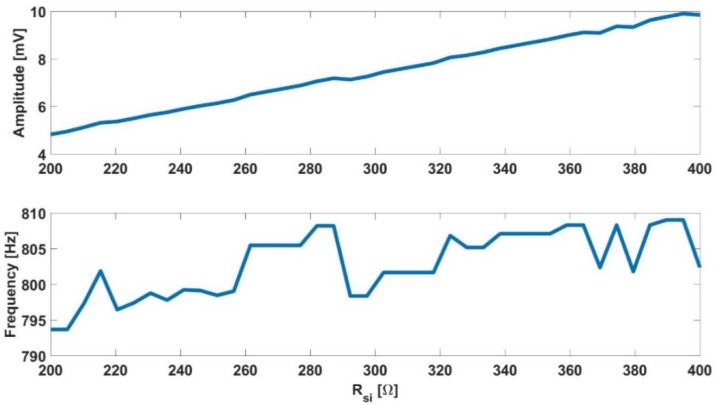
Variation of initial oscillation amplitude and frequency as a function of *R_s_* value.

**Figure A4:**
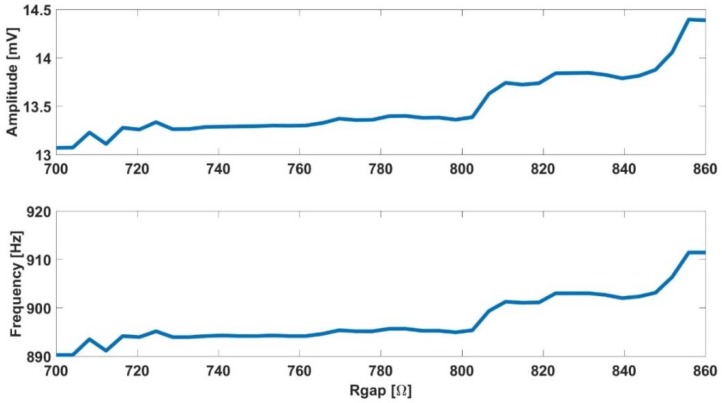
Variation of final oscillation amplitude and frequency as a function of *R_gap_* value.

**Figure A5:**
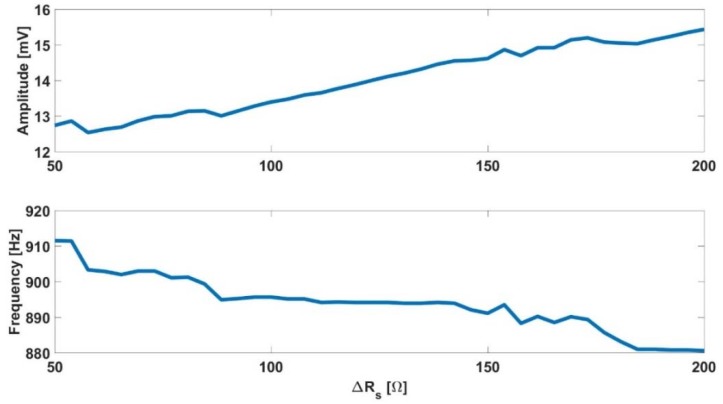
Variation of final oscillation amplitude and frequency as a function of Δ*R_s_* value.

Figure A2, Figure A3, Figure A4 and Figure A5 show the changes of the oscillation frequency and amplitude at the beginning and at the end of the experiment with the model parameters ($f_{p}$, $R_{s}$, $R_{gap}$ and $\Delta R_{s}$). In following section will explain how the OBT works, and the equations involved. These figures have been obtained simulating the OBT system by MultiSim, for limit values of ff→0 (initial conditions) and ff→1 (final conditions), varying the parameters in the range that they usually work with.

Figure A2 displays how the initial oscillation amplitude and frequency (ff→0) depends on the frequency of the pole. Figure A3 illustrates that, after choosing the pole position at $f_{p}=95\;\mathrm{Hz}$, the initial oscillation amplitude changes with $R_{s}$, without significant changes in the initial oscillation frequency. In Figure A4 and Figure A5 it can be seen that the final oscillation amplitude and frequency (ff→1) depends on $R_{gap}$ and $\Delta R_{s}$ (a parameter that is included in the model as explained below).

If we try to fit the experimental results with the BioZ electrical model, we cannot reach the full oscillation amplitude range, so it is necessary to introduce a small variation. To solve this problem, we firstly tried to follow the work in [9], making a correction of $R_{s}$ with *ff*. Thus, we considered $R_{s}$ when ff→0 is an initial value of $R_{s}$ called $R_{si}$. Then it is necessary to increase (or decrease) $R_{s}$ with *ff* during the experiment until the experiment is over, and at that moment (ff→1) $R_{s}$ will reach the value for which the final model amplitude will be similar to the final experiment amplitude. This is achieved by varying $R_{s}$ with the Equation (5).

(5) $$R_{s}\left(k\right)=R_{si}+\Delta R_{s}\times ff{\left(k\right)}^{n}$$

where *k* is the time index of experiment, $R_{si}$ is initial value for $R_{s}$, $\Delta R_{s}$ is the range of $R_{s}$ (from *ff* = 0 to *ff* = 1). *ff*(*k*) is the fill-factor at *k*, and *n* is the growth rate of $R_{s}$ from $R_{si}$ to $R_{si}+\Delta R_{s}$.

To quantify the improvement introduced by the correction of $R_{s}$, we observed the relative error of the amplitude with respect to the experimental results when *ff* → 1. The relative error before $R_{s}$ correction was 7.9%, while the relative error after $R_{s}$ correction was 1.5%. Therefore, the improvement of $R_{s}$ correction was remarkable and necessary to make a good fit for the BioZ model.

**Figure 2:**
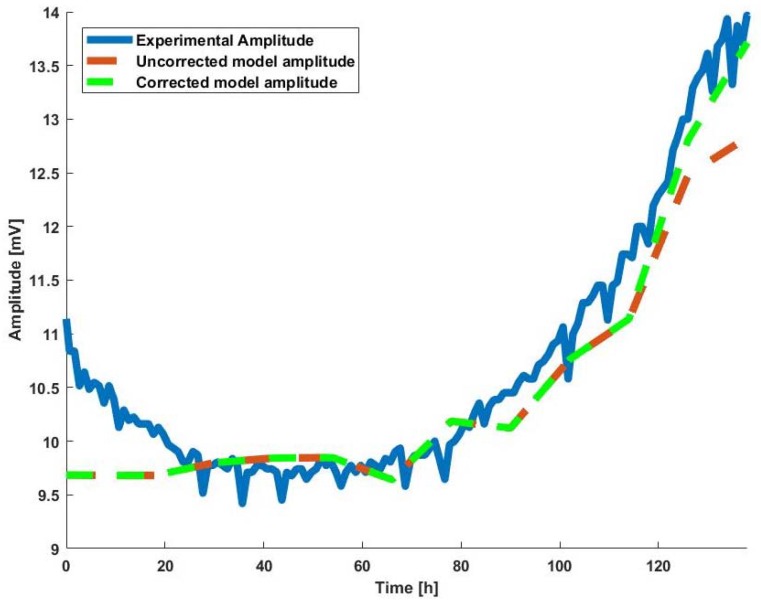
Comparison between the experimental amplitude, the amplitude of the uncorrected *R_s_* electric model, and the amplitude of the *R_s_*-corrected electric model.

Finally, Figure 2 shows the experimental amplitude evolution (blue) and the evolution of the oscillation amplitude of the fitting model without $R_{s}$ correction (red). As can be seen, there is a certain error, and by applying the correction to $R_{s}$, it is possible to reduce the error almost completely (green).

### 2.4. On-Line Estimation

#### 2.4.1. System Equations

**Figure 3:**
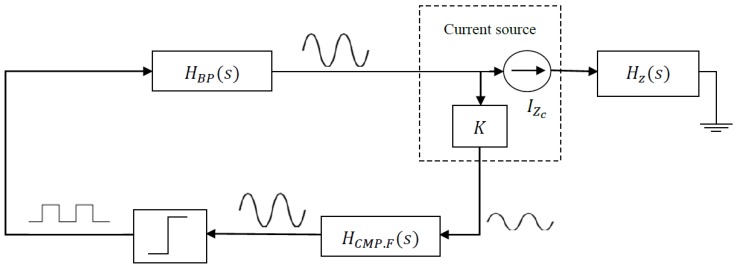
Oscillation-based test (OBT) block diagram.

A mathematical description of the full system was required for cell-electrode electrical model estimation. The circuit system described in Figure 3 [15,16,17,18] has three main circuit blocks; a band-pass filter (*H_BP_*), a current source (*I_Zc_*), and a voltage comparator block (*K*-gain amplifier, comparator filter – *H_CMP,F_* –, and the voltage discriminator).

As previously mentioned, to estimate the concentration of cells at each moment of the experiment, several points must be known:

○ The equations of entire system; that is, the equations of the BioZ electric model and the oscillator. From these equations and using the Describing Function method [20], two equations are obtained whose solutions allow us to derive the oscillation amplitude and frequency.
○ The BioZ equations:
  (6) $$H_{z}\left(s\right)=\frac{k_{2}\times s^{2}+k_{1}\frac{\omega_{0z}}{Q_{z}}s+k_{0}\omega_{0z}^{2}}{s^{2}+\frac{\omega_{0z}}{Q_{z}}s+\omega_{0z}^{2}}$$
  where $H_{z}\left(s\right)$ is the transfer function of the cell-electrode electrical model and the other parameters are defined by the resistances and the capacitors of the electric model ($R_{s}$, $R_{gap}$, $R_{1}$, $R_{2}$, $C_{1}$ and $C_{2}$), as can be seen on Equations (7)–(11).
  (7) $$k_{2}=R_{s}$$
  (8) $$k_{1}=R_{s}+\frac{R_{gap}R_{1}}{2R_{gap}+R_{1}+R_{2}}$$
  (9) $$k_{0}=R_{s}+\frac{R_{1}\left(R_{gap}+R_{2}\right)}{R_{gap}+R_{1}+R_{2}}$$
  (10) $$\omega_{0z}=\sqrt{\frac{R_{gap}+R_{1}+R_{2}}{R_{gap}{\left(RC\right)}^{2}}}$$
  (11) $$Q_{z}=\omega_{0}\frac{R_{gap}RC}{2R_{gap}+R_{1}+R_{2}}$$
○ The OBT equations (see Figure 3 and [15,16]):
  (12) $$H_{BP}\left(s\right)=\frac{K\frac{\omega_{0}}{Q}\times s}{s^{2}+\frac{\omega_{0}}{Q}\times s+\omega_{0}^{2}}$$
  (13) $$\begin{array}{l} H_{CMP.F}\left(s\right)\\ =\frac{k_{h}\times k_{l}\times s^{2}}{s^{4}+\left(\frac{\omega_{0h}}{Q_{h}}+\frac{\omega_{0l}}{Q_{l}}\right)s^{3}+\left(\omega_{0h}^{2}+\omega_{0l}^{2}+\frac{\omega_{0h}\omega_{0l}}{Q_{h}Q_{l}}\right)s^{2}+\left(\frac{\omega_{0h}\omega_{0l}^{2}}{Q_{h}}+\frac{\omega_{0l}\omega_{0h}^{2}}{Q_{l}}\right)\times s+\omega_{0h}^{2}\omega_{0l}^{2}} \end{array}$$
  (14) $$N\left(a_{osc}\right)=\frac{4V_{ref}}{\pi a_{osc}}\times \left(-\cos\theta +\sin\theta \right)\;\;\;\theta =\sin^{-1}\left(h/a_{osc}\right)$$
  (15) $$H_{Z.CS}\left(s\right)=k_{z}H_{z}\left(s\right)$$

Equations (12)–(15) are the functions which describe the OBT approach (Figure 3). Equation (12) is the transfer function of the required band-pass filter, whose natural frequency is $\omega_{0}$ and *Q* it is its quality factor. Equation (13) is the transfer function of a filter, composed of a low pass filter and a high pass filter, whose natural frequencies are $\omega_{0l}$ and $\omega_{0h}$, respectively, and $Q_{l}$ and $Q_{h}$ are their respective quality factors. On the other hand, $N\left(a_{osc}\right)$ in Equation (14), is the linearized transfer function of the comparator obtained from the Describing Function method [20]. In this equation, $V_{ref}$ is the reference voltage of the comparator, *a_osc_* is the oscillation amplitude of the input signal of the comparator, and *h* is a parameter that depends on the hysteresis of the comparator (which is configured through a feedback of passive components). Finally, *H_Z.CS_*(*s*) is the transfer function of the current source to which the BioZ is connected as a load. The transfer function of BioZ is multiplied by a constant that depends on the resistances of the current source.

The Barkhausen stability criterion (16) is the mathematical condition that the closed loop feedback system (Figure 3) has to accomplish in order to obtain sustained oscillations. Thus, the oscillation parameters, *a_osc_* and *f_osc_*, can be derived by equating the real and imaginary part of Equation (16) to 0, where $\omega =2\pi f_{osc}$.

(16) $$1+H_{BP}\left(s=j\omega \right)\times H_{Z.CS}\left(s=j\omega \right)\times H_{CMP.F}\left(s=j\omega \right)\times N\left(a_{osc}\right)=0$$

○ The parameters of the BioZ electric model, which are obtained from calibration (as will be discussed below), are incorporated to Equation (16), introducing the electrode parameter influence on solutions derived for amplitude and frequency.
○ The relationship between *ff* and the number of cells, which will depend on the maximum area to be covered by the cells and the cell size for each cell line.

#### 2.4.2. System Calibration

The system calibration is very important since it is the method to know the values of the BioZ model parameters. The selected calibration protocol was as follows:

1. The first step (for the calibration experiment) was to perform an initial experiment to estimate $R_{gap}$ and $\Delta R_{s}$ (the measurements of two wells are used; that is, two cell cultures with the same initial conditions). These parameters not only defined the oscillation frequency and amplitude when the well is full, but also defined the range of oscillation frequency and amplitude from ff→0 to ff→1. As these parameters could only be estimated when the culture reached the confluent or mono-layer phase, and the range of frequency and amplitude for the same cell line did not vary much (unlike the initial frequency and amplitude of oscillation), it was decided that these two parameters, $R_{gap}$ and $\Delta R_{s}$, were common to the cell line. This first experiment was only done once per cell line.

**Figure 4:**
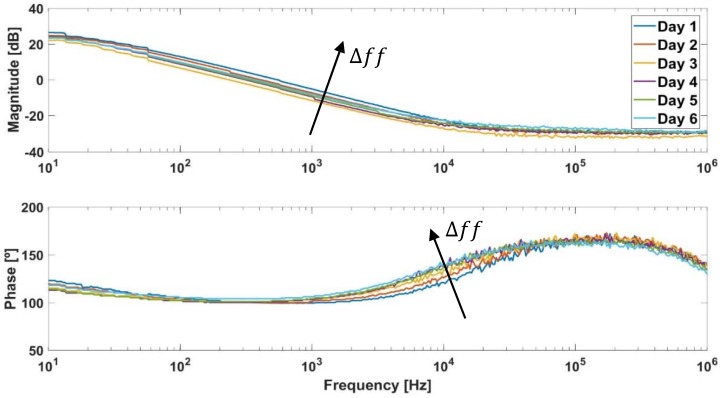
Experimental BioZ bode diagram.

**Figure A6:**
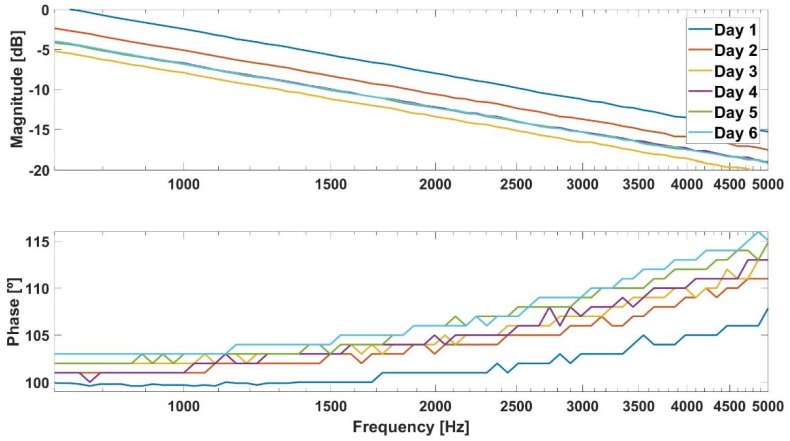
Area of interest in the Bode diagram from Figure 4.

Both the system equations (OBT) and the equations of BioZ electric model ((6) and (16), respectively) were used to obtain $R_{gap}$ and $\Delta R_{s}$. The position of the zero of the BioZ model was fixed at 15 kHz for all experiments, since it was verified by means of the analysis of the Bode diagram of the cell-electrode system (in several experiments) that was always located at this frequency, as can be seen in Figure 4 (areas of the Bode diagram of the frequencies of interest in our system are shown in Figure A6). From the frequency and amplitude of oscillations at the beginning of the experiment (ff→0), it is known that the value of $R_{gap}$ has an almost null influence at this point of the experiment and considering that by definition in Equation (5) $\Delta R_{s}$ is 0, and $f_{p}$ and $R_{si}$ can be obtained for the calibration experiment. After that, $R_{gap}$ and $\Delta R_{s}$ are achieved using the oscillation amplitude and frequency when ff→1, which are the same in each cell line.

2. Second step (for the real-time measured experiment): The currently used electrodes did not present great precision at the beginning of the experiment, since in each well, the cells could be deposited in different positions, and the distribution of the electrodes in the wells was not optimal. Therefore, at the beginning of each experiment, knowing the oscillation amplitude and frequency, the pole position ($f_{p}$) and $R_{si}$ could be obtained in the same way as in the calibration experiment, and in this case they would be unique for each well. This step had to be done in order to reach the best estimation of *ff*, and it does not suppose loss of information, since they are obtained in the early hours of the experiment. Any current CPU (Central Processing Unit) takes very little time to perform this task, and this time is significantly smaller than the experiment sampling time (1 h).

Therefore, the calibration is completed when all these steps are done in such a way that *ff* can be obtained in real time for each experiment of a cell line.

#### 2.4.3. *ff*-Number of Cells Relationship

The *ff* may not be useful in order to know parameters like birth and mortality rate, growth rate, etc. of a cell culture. However, it would be suitable to know the number of cells in each well in real time. There is a direct relationship between *ff* and number of cells, which depends on well area ($A_{p}$) and area of each single cell. This relationship is provided by Equation (17):

(17) $$ff=\frac{A_{cell}}{A_{p}}\times N_{cell}$$

where *ff* is the percentage of well filling, *N_cell_* is the number of cells for a value of *ff* and $A_{cell}$ is the average area of cells in each cell line. The well area is known (provided by the manufacturer [19]), whereas the cell area is unknown. The growth curve of each cell line (cell density vs time) obtained by conventional methods is used to estimate the cell area of each cell line. The number of cells in the confluent phase matches with the moment when *ff* is near to 1, so Equation (17) can be used to find the cell area of each cell line. The average area of each studied cell line is in Table 1.

## 3. Results

### 3.1. Experimental Cell-Line ff Curves

For each cell line, one experiment was carried out with an initial number of cells of 10,000 cells (Section 2.1). This first experiment was used for calibration; in other words it, was used to obtain the parameters that define the ranges of oscillation amplitude and frequency during the experiments. These ranges could only be obtained when the experiment was over. These parameters were estimated using the system equations, and the oscillation amplitude and frequency when the cell culture was at the monolayer phase (ff→1). $R_{s}$ changed because, as the number of cells in the cell culture increased, the size of the cell layer increased; therefore the path from the double layer cell-electrode to the reference electrode changed. For this reason, $\Delta R_{s}$ could be negative.

Table 2 shows $R_{gap}$ and $\Delta R_{s}$ (it can be negative if the condition $R_{si}+\Delta R_{s}\geq 0$ is met) for each cell line, which will be used for the real-time estimation of *ff* in the subsequent experiments.

**Figure 5:**
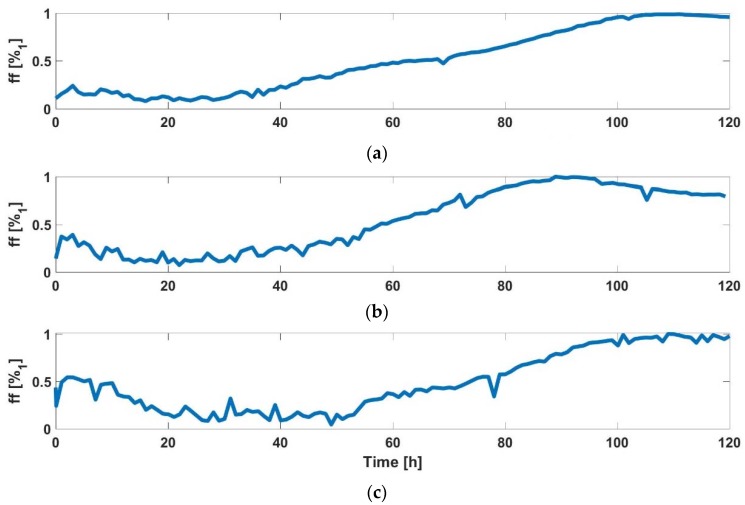
Estimated *ff* for: (**a**) AA8 cell line model, (**b**) N2aAPP cell line model and (**c**) N2A cell line model.

For each cell line, *ff* was estimated using the electrical model of BioZ (obtained in the calibration phase) and the experimental oscillation amplitude and frequency. In this way, we could make a first test of the model. Figure 5 shows the evolution of *ff* for each cell line in the calibration experiment.

Each cell line had a different evolution and range of oscillation amplitude and frequency, so it was important to characterize it in the first experiment. After that, any culture assays could be monitored in real time, either in an experiment of cell growth, toxicity of radiation, etc.

A real time experiment was simulated using a Matlab script (from data of other experiments) to test and validate our method of adjustment and monitoring. The first step in this experiment was, taking into account the initial amplitude and frequency of oscillations after the initial transient (which is different for each cell line), to calculate the pole location ($f_{p}$) and $R_{s}$ when ff→0 ($R_{si}$). After some initial measurements, both the system equations and the BioZ electrical model were used to estimate $f_{p}$ and $R_{si}$. Thus, with $R_{gap}$ and $\Delta R_{s}$ obtained in the calibration experiment, we generated a rough estimate of *ff* for each oscillation amplitude and frequency value.

Some simulations of real time experiments were carried out, as was explained in the previous paragraph. These simulations were made using experiment data with different initial cell densities to corroborate the method. Even so, there is an *ff* estimation error that was adjusted at the end of the experiment. Error correction was done to estimate the error and validate this technique. *ff* is corrected using Equation (18).

(18) $$ff_{cor}\left(k\right)=ff\left(k\right)\times \frac{ff_{max.ideal}-ff_{min.real}}{ff_{max.real}-ff_{min.real}}$$

where $ff_{cor}\left(k\right)$ is *ff* corrected at the instant *k*, ff(k) is *ff* without correction at the instant *k*, $ff_{max.ideal}$ is the ideal value of *ff* in the confluent phase (0,99), $ff_{max.real}$ is the maximum value of *ff* obtained in the real time experiment, and, $ff_{min.real}\;$ is the minimum value of *ff* obtained in the real time experiment. Thus, *ff* is corrected according to its real value at the beginning of the experiment, which is considered to be the real value because an actual $f_{p}$ and an actual $R_{si}$ have been obtained for each well at the beginning of the experiment.

**Figure 6:**
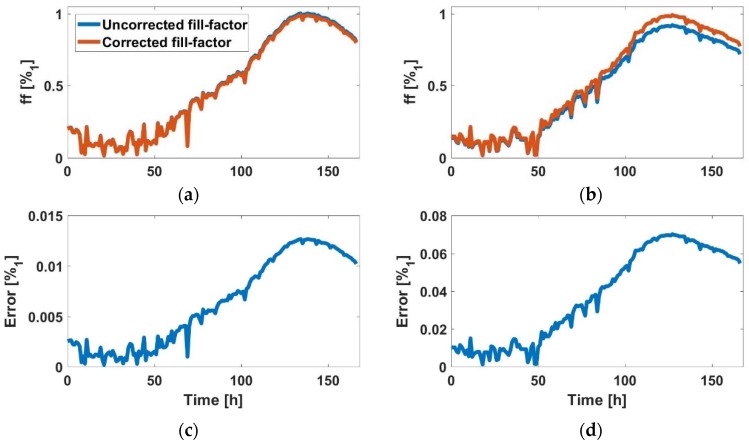
(**a**) Comparison between uncorrected and corrected *ff*, and (**b**) *ff* estimation error (AA8 with 2500 cells at the beginning). (**c**) Comparison between uncorrected and corrected *ff*, and (**d**) *ff* estimation error (AA8 with 5000 cells at the beginning).

**Figure 7:**
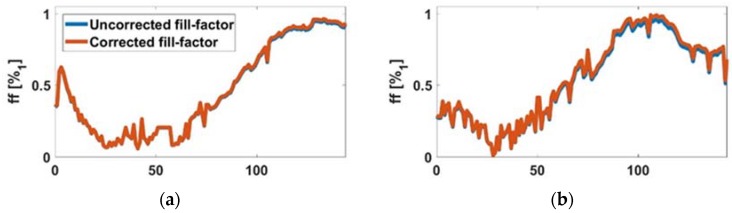

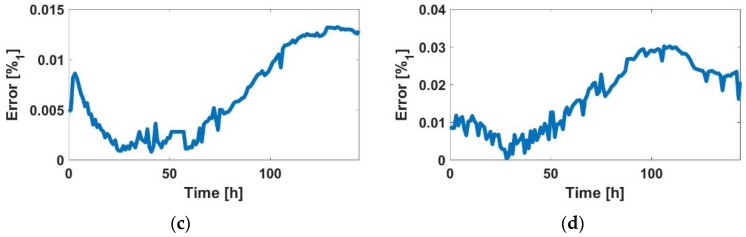
(**a**) Comparison between uncorrected and corrected *ff*. (**b**) *ff* estimation error (N2aAPP with 2500 cells at the beginning). (**c**) Comparison between uncorrected and corrected *ff*, and (**d**) *ff* estimation error (N2aAPP with 5000 cells at the beginning).

**Figure 8:**
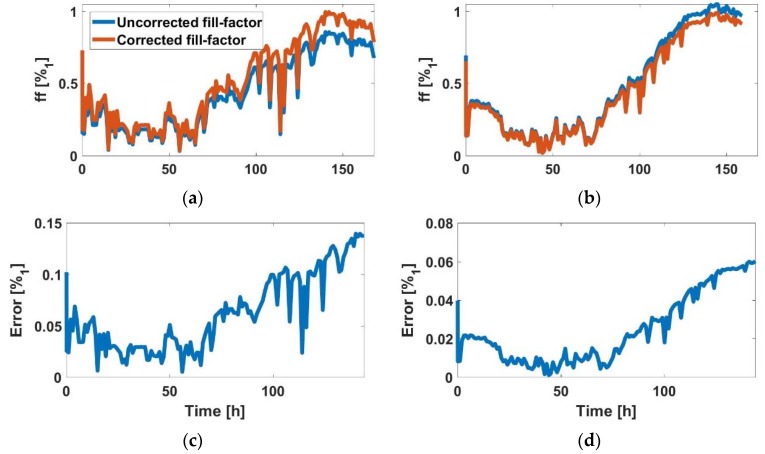
(**a**) Comparison between uncorrected and corrected *ff*, and (**b**) *ff* estimation error (N2a with 2500 cells at the beginning)**.** (**c**) Comparison between uncorrected and corrected *ff,* and (**d**) *ff* estimation error (N2a with 5000 cells at the beginning).

For the AA8 cell line, in Figure 6a (2500 cells at t=0 h) and Figure 6c (5000 cells at t=0 h), the graphics shows the estimated *ff* during the experiment (blue) and after correction at the end of experiment (red). In Figure 6b,d the absolute error in both experiments, which was small, is shown. Hence the *ff* estimation method was validated, since maximum calculated *ff* approached well to unity. In the same way, the N2aAPP cell line, in Figure 7a (2500 cells at t=0 h) and Figure 6c (5000 cells at t=0 h), the estimated *ff* is illustrated during the experiment (blue), and after correction at the end of experiment (red), respectively. In Figure 7b,d, the absolute error in both experiments was small, being below 3%. Finally, for the N2a cell line, in Figure 8a (2500 cells at t=0 h) and Figure 6c (5000 cells at t=0 h), the estimated *ff* during the experiment (blue) and after correction at the end of experiment (red) is shown. The maximum absolute error (Figure 8b,d) in both experiments was below 14%, which was the largest error observed for the three cell lines.

### 3.2. Experimental Cell-Line Growth Curves

Finally, to obtain the concentration of cells in each well, Equation (17) was applied. The cell density error was similar to the *ff* error, which was corrected at the end of experiment. The error was as small as the uncorrected *ff* error, and was acceptable. In any case, at the end of experiment, an accurate cell density was obtained using the corrected *ff*.

**Figure 9:**
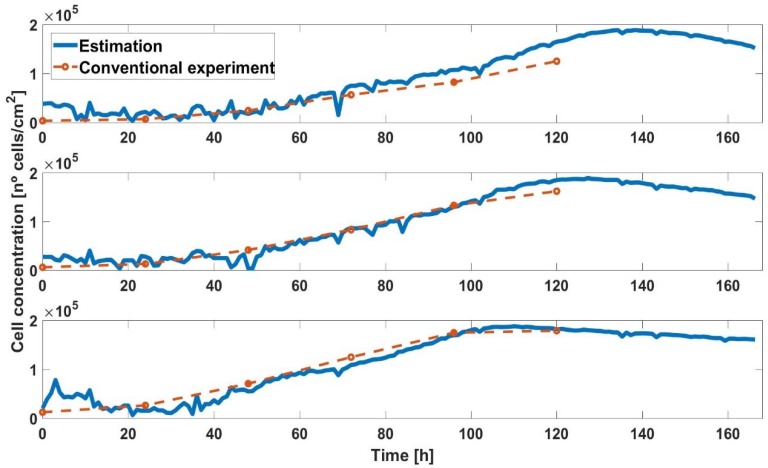
Growth curve with initial number of cells 2500, 5000, and 10,000 (AA8).

In Figure 9, the growth curves of AA8 cell line are compared. Blue lines are the cell density estimated from the corrected *ff*, and red lines are the growth curves obtained from standard methods with petri plates. These curves showed that the evolution of the cell densities, in both case, matched well for the three initial concentrations of AA8 cells. It was employed in a cell area shown in Table 1.

Firstly, if we focused on the first 20–24 h of the experiment, we observed that the cells needed that time (depending on the cell line and the type of medium) to settle into the well. From here on, both curves were very similar, but we should keep in mind that traditional curves are made by counting and statistically, so there may have been some error.

We did not complete the experiments until the cell culture assay was in the death phase, in order to collect as much data as possible. To have a reference, the curves made by traditional methods must have a similar length.

## 4. Discussion

The obtained results for AA8 cell line are good enough to consider the proposed method for calibration and cell growth estimation as a real contribution in the field of electric modelling of cell-electrode system, with application in cell culture assays. However, some points must be clarified about the presented work. Firstly, the estimation of the attained initial cell concentrations is always larger than expected. This can be due to several factors. When cells are seeded, they spend some time adapting to the environment. This effect is filtered by classical techniques, which take out the first count at 24 h, after the adaption processes has finished. In our approach, the sample rate is 1 h, so this effect is fully observed by the electrodes of the measurement system. Even more, the cell density at the beginning is low, so electrode area could not sense the cells if they were not on the top. By increasing the sensing surface (electrode number and area) this effect should be reduced. Secondly, the estimated cell size has been considered constant along the cell culture assay. This could be not true, because the cell size, and the corresponding cell-to-electrode interface, could be reduced when the cell density increases in time. These smaller cell sizes can lead to an underestimation of the cell number. As well, the cell lines with the same initial cell density will have different system responses as a consequence of the cell size specifics of each cell line, since the covered substrate area is different. At the initial time, cell-to-cell distance will be also different depending on the cell line, for the same initial cell density. Thirdly, the reason for a negative increment on spreading resistance in the N2a cell line electrical model is not clear, and the authors are not sure if it is a real physical effect or if it was derived from the calibration process. This spreading resistance models the conductivity from working electrode to reference electrode along the culture medium, and other parameters could potentially influence it, such as the barrier resistance, and these should be incorporated into the proposed electrical model in the future. Finally, the proposed calibration method delivers some parameters (*R_gap_* and Δ*R_s_*) that can be considered representative of each cell line, of course considering that the specific well-electrode system has been employed for assays and that the obtained results will depend on the possibility of developing a possible method for cell biometry, a difference of other approaches that only measures the final impedance of the cell cultures.

## 5. Conclusions

Using the proposed calibration protocol and the OBT technique, an accurate cell-electrode electrical model useful for the bioimpedance ECIS technique experiments can be obtained for any cell line. Both the BioZ electric model described and the OBT technique allow the estimation of the well filling percentage (*ff*), with relatively low errors. Likewise, if the cell area is known, the cell density value is predicted in real time. The proposed calibration and fitting technique can be also applied to any other circuit technique for electrical cell-substrate impedance spectroscopy measurements.

Thanks to the correction proposed for the spreading resistance parameter, $R_{s}$, and the calibration protocol described, the obtained electric model of the cell-electrode system is improved by reducing its error near the confluence phase. In summary, the cell number in a culture can also be accurately monitored in real time.

In this work, Matlab software has been used to simulate the calibration protocol, and for the testing and validation of the model in real time. The next step is to implement the Matlab script in the smart prototype of our OBT system approach [15,16,17,18]. The OBT prototype will then be able to autonomously follow the *ff* and the cell density in real time.

## Figures and Tables

**Table 1:** Cell area of the studied cell lines.

Cell Line	$A_{cell}\;\left[\mu m^{2}\right]$
AA8	531
N2aAPP	118
N2a	184

**Table 2:** $R_{gap}$ and $\Delta R_{s}$ for each cell line.

Cell Line	$R_{gap}\;[\Omega ]$	$\Delta R_{s}\;[\Omega ]$
AA8	889	102
N2aAPP	572	21
N2a	360	−19
